# Supplementary material for: Tuning orbital orientation endows molybdenum disulfide with exceptional alkaline hydrogen evolution capability
Source: Nat Commun. 2019 Mar 14;10:1217. doi: 10.1038/s41467-019-09210-0 (PMC6418089; doi:10.1038/s41467-019-09210-0)
Supplement: Supplementary file 1 — Supplementary Information [file 41467_2019_9210_MOESM1_ESM.pdf]

Supplementary Information

Tuning orbital orientation endows molybdenum disulfide with exceptional alkaline hydrogen evolution capability

Zang et al

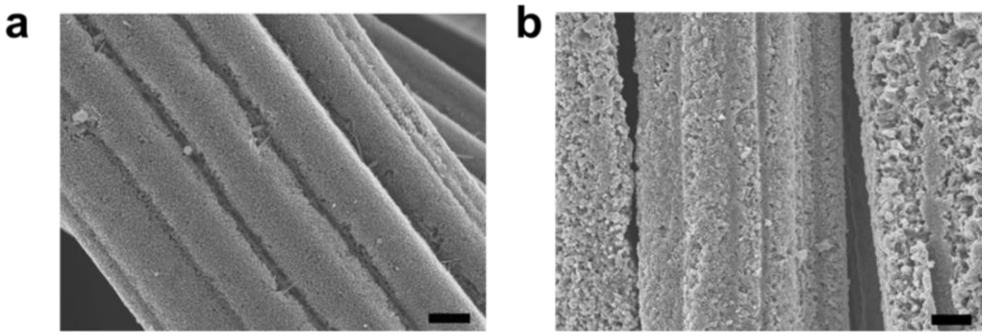

Supplementary Figure 1. Morphology characterization. SEM images of (a) Mo_2C and (b) C-MoS_2. Scale bars, 1μm.

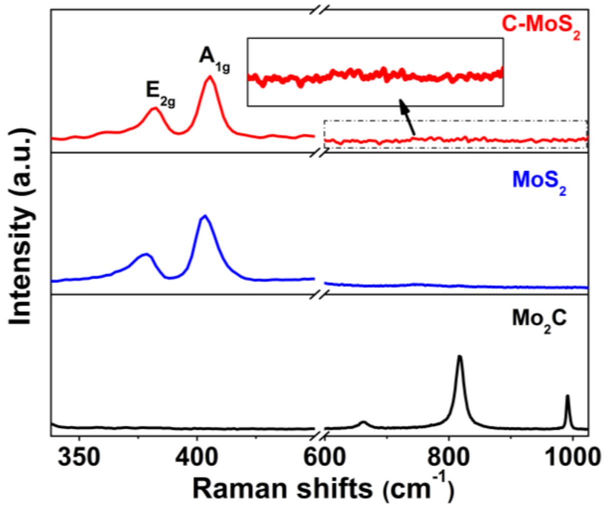

Supplementary Figure 2. Raman spectroscopy analysis. Raman spectra of Mo_2C, MoS_2 and C-MoS_2. The inset is the magnified region highlighted by the dashed box.

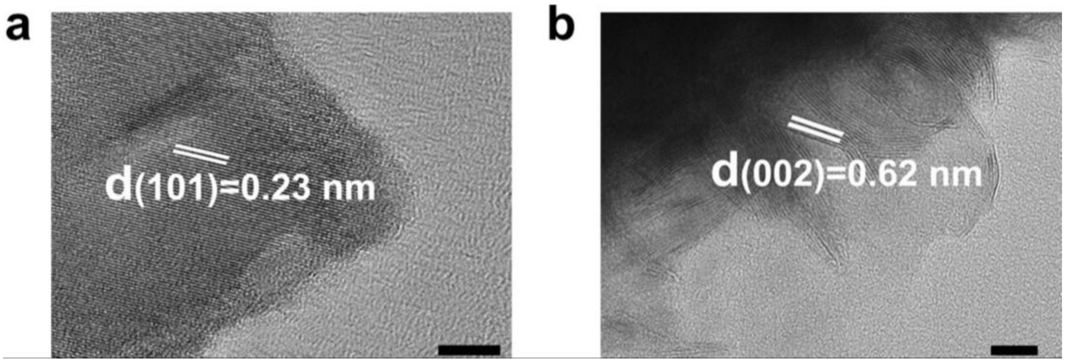

Supplementary Figure 3. Transmission electron microscopy characterization.

TEM images of (a) Mo_2C, the scale bar is 5 nm and (b) MoS_2, the scale bar is 10 nm.

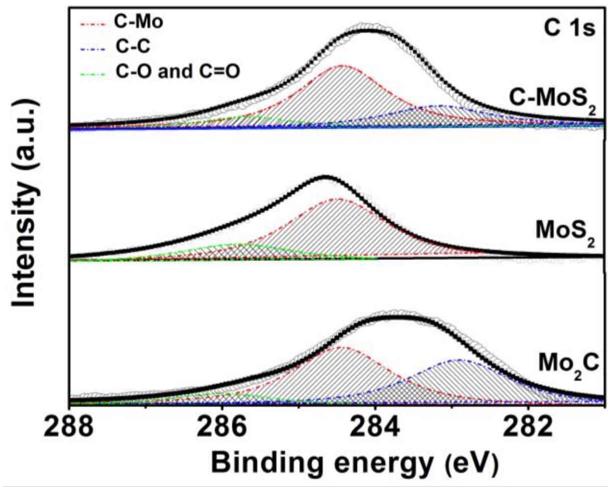

Supplementary Figure 4. XPS C1s spectra analysis. XPS core-level spectra of C 1s for Mo_2C, MoS_2 and C-MoS_2.

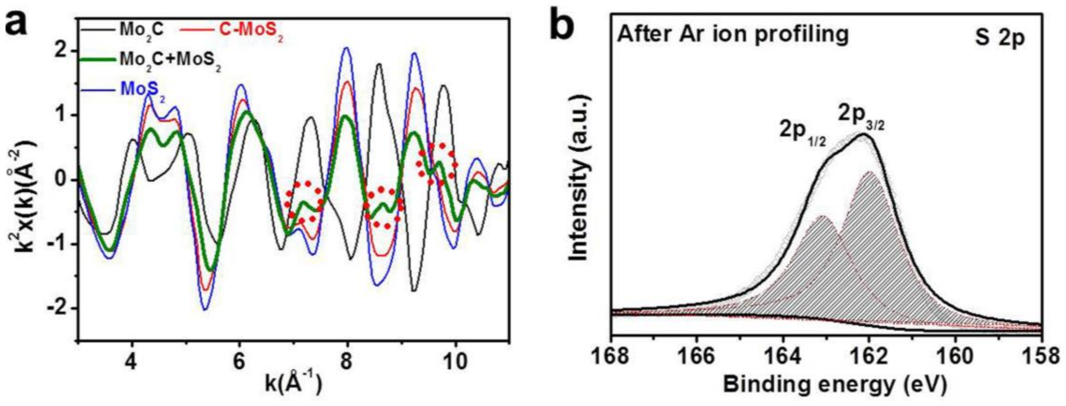

Supplementary Figure 5. Extended XAFS oscillation functions and XPS S 2p spectra. (a) The Mo k-edge extended XAFS oscillation functions $k^2\chi(k)$ of MoS_2, C-MoS_2, Mo_2C and the physical mixture of Mo_2C and MoS_2. (b) XPS S 2p spectrum of C-MoS_2 after deep Ar ion profiling.

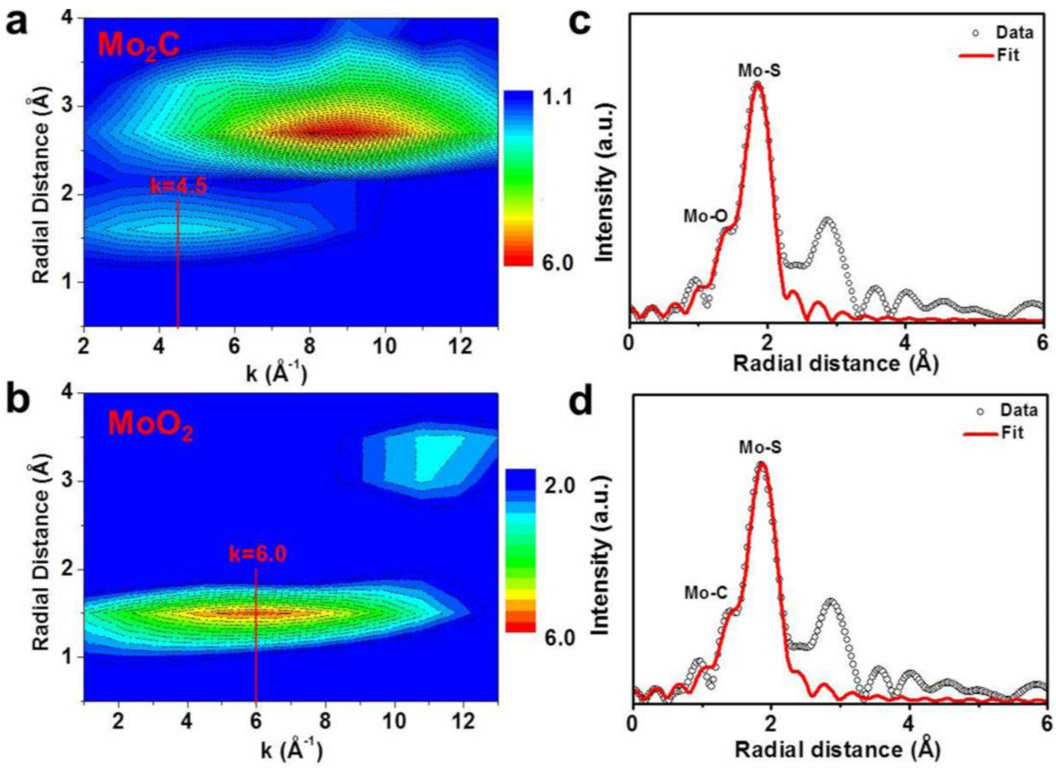

Supplementary Figure 6. Wavelet transform and radial distribution of Fourier-transformed EXAFS. The whole contour plots of the wavelet transform (WT) of Mo_2C (a) and MoO_2 (b). Radial distribution of Fourier-transformed EXAFS signal of C-MoS_2 with a Mo-O path (c) and Mo-C (d) at R = 1.3 Å.

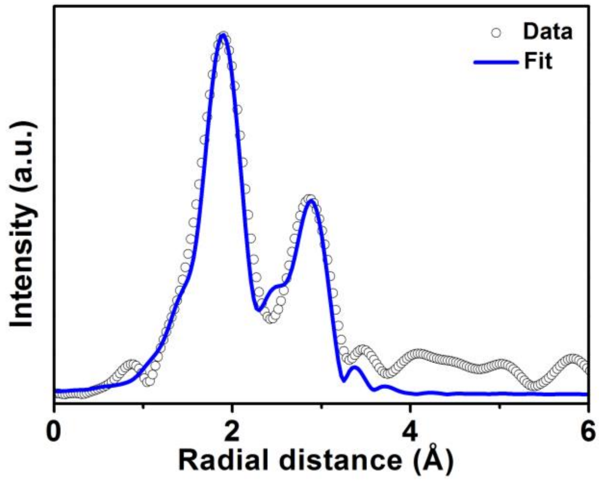

Supplementary Figure 7. Radial distribution analysis. Radial distribution of Fourier-transformed EXAFS spectrum of MoS_2.

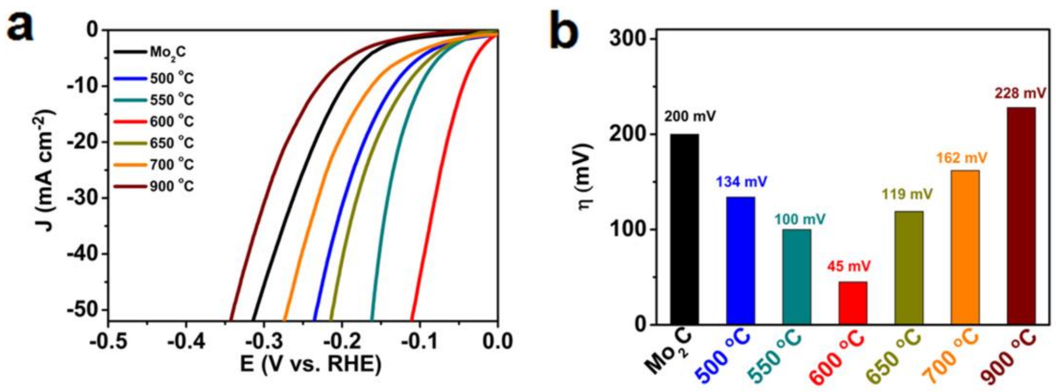

Supplementary Figure 8. The temperature-dependent HER performance of C-MoS$_2$. (a) LSV curves of Mo_2C and C-MoS$_2$ obtained at different sulfurization temperatures with a scan rate of 5 mV s^{-1} in 1.0 M KOH solution. (b) The corresponding overpotentials at 10 mA cm^{-2}.

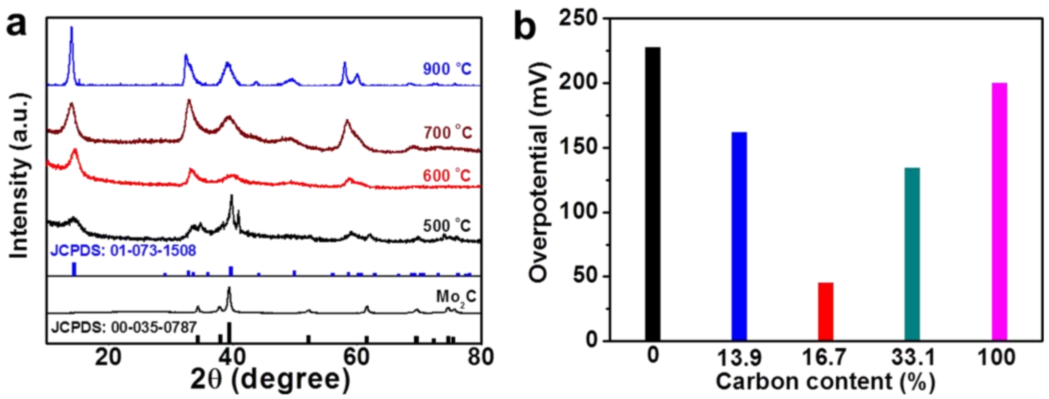

Supplementary Figure 9. Temperature-dependent XRD patterns and carbon-dependence HER performance. (a) The XRD patterns of Mo_2C and C-MoS_2 obtained at different sulfurization temperatures. (b) The carbon content dependent HER activities.

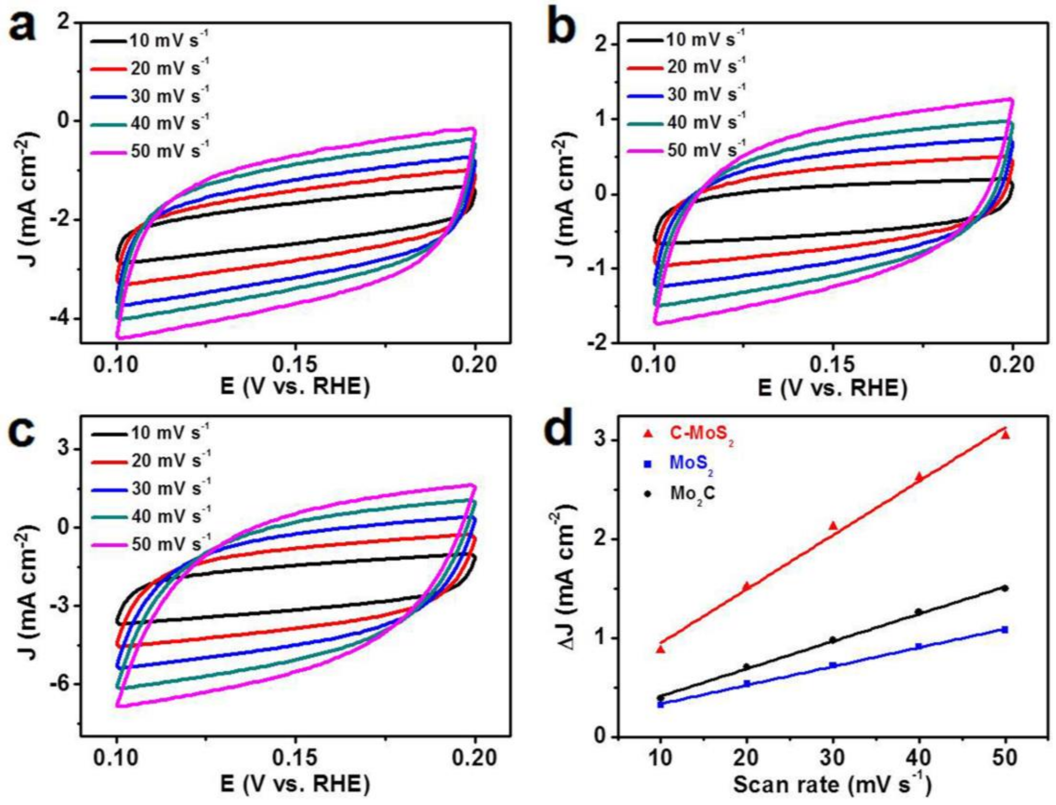

Supplementary Figure 10. Electrochemical surface area analysis. CV curves at different scan rates from 10 to 50 mV s^{-1} of (a) Mo_2C, (b) MoS_2 and (c) C-MoS_2. (d) The plots of ΔJ versus scan rates for the Mo_2C, MoS_2 and C-MoS_2, respectively.

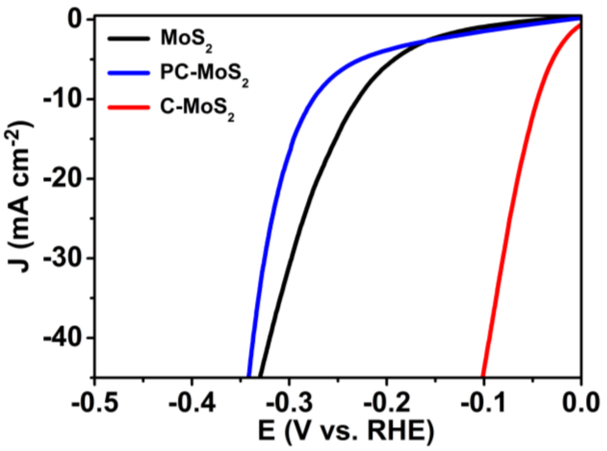

Supplementary Figure 11. The HER performance comparison of carbon doped MoS_2 with different preparation methods. LSV plots of MoS_2, C-MoS_2 and PC-MoS_2 with a scan rate of 5 mV s^{-1} in 1.0 M KOH solution.

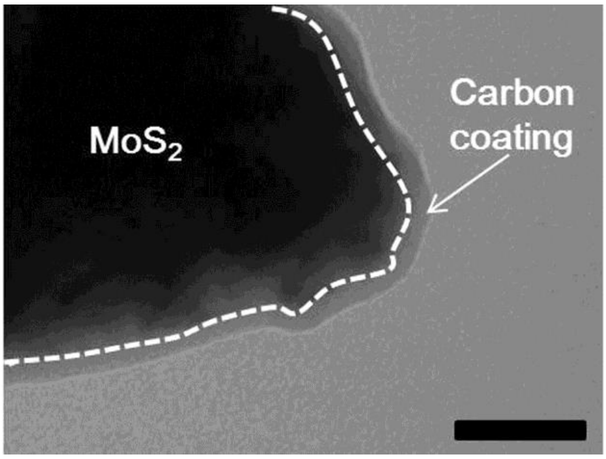

Supplementary Figure 12. Transmission electron microscopy analysis of PC-MoS_2. TEM image of PC-MoS_2, with carbon shell highlighted by dash lines. The scale bar is 50 nm.

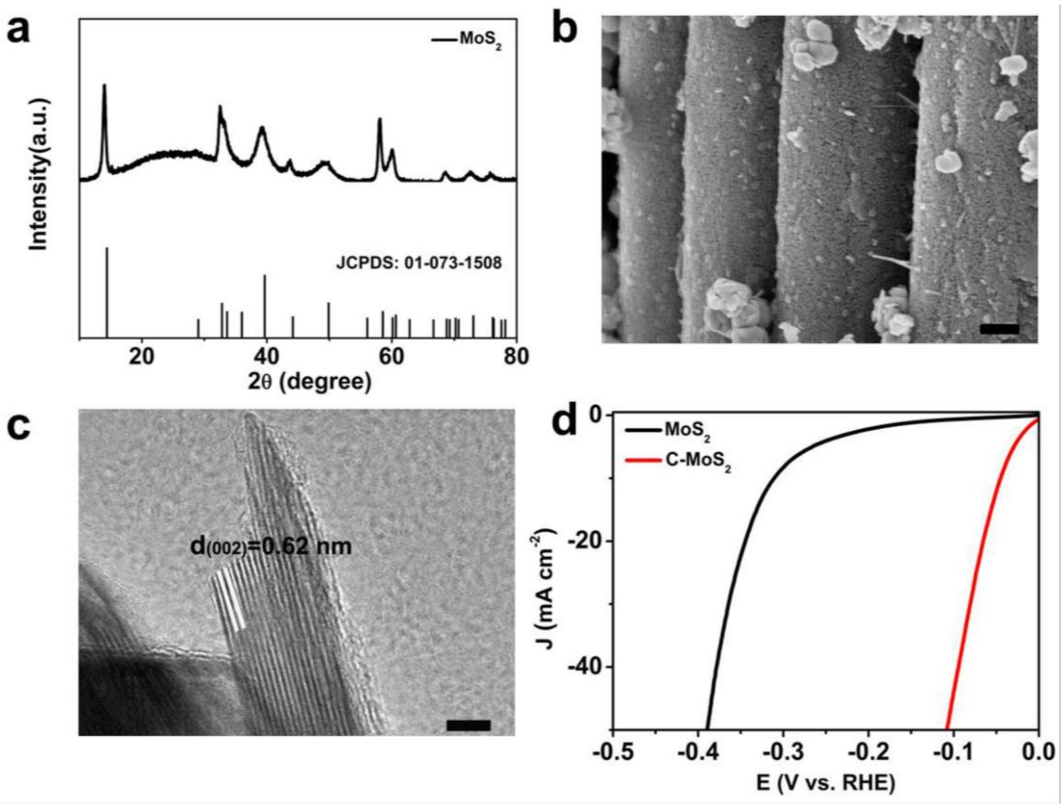

Supplementary Figure 13. Crystal structure, morphological characterization and HER analysis of MoS_2 without carbon involvement. (a) XRD pattern, (b) SEM (the scale bar is 1 μm) and (c) HRTEM (the scale bar is 5 nm) images of the MoS_2 synthesized by thermal sulfurization of ammonium molybdate with S powder as the sulfur source. (d) The LSV curves of the C-MoS_2 and the MoS_2 for alkaline HER catalysis.

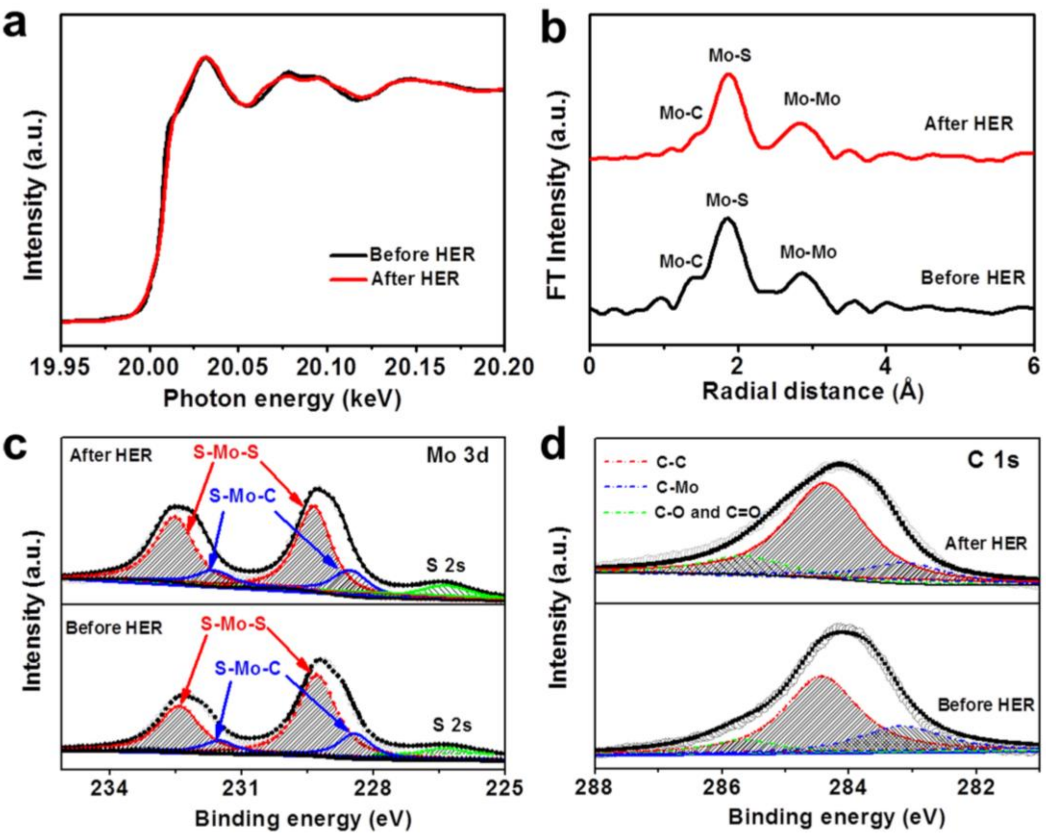

Supplementary Figure 14. The chemical states of C-MoS_2 before and after the HER durability test. (a) The normalized Mo K-edge XANES spectra. (b) The Fourier transform (FT) of Mo K-edge. XPS core-level Mo 3d (c) and C 1s (d) spectra of the C-MoS_2 before and after the durability test.

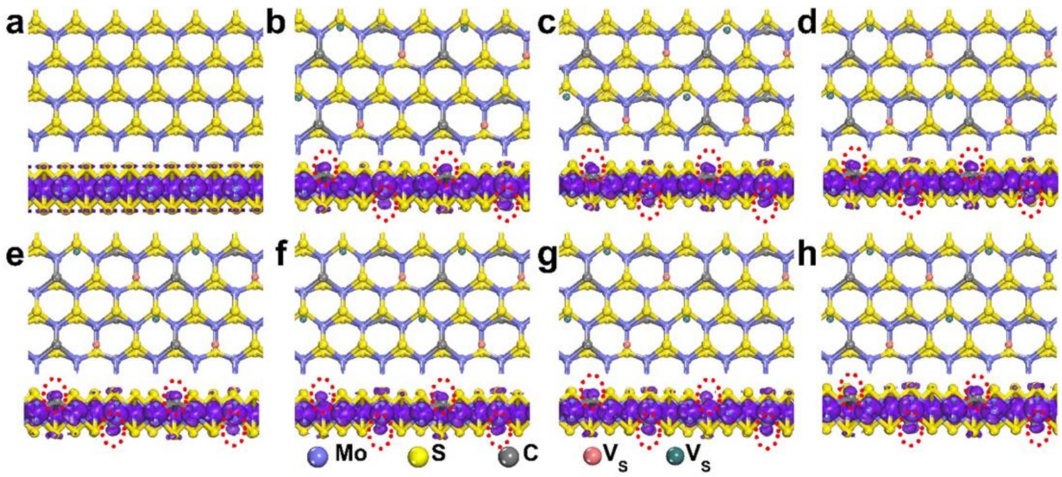

Supplementary Figure 15. **Structure and orbital analysis.** The top views (upper) of MoS_2 and C-MoS_2 structures, and the corresponding side view (lower) of empty orbital orientations with different distributions of the carbon dopants and involved vacancies.

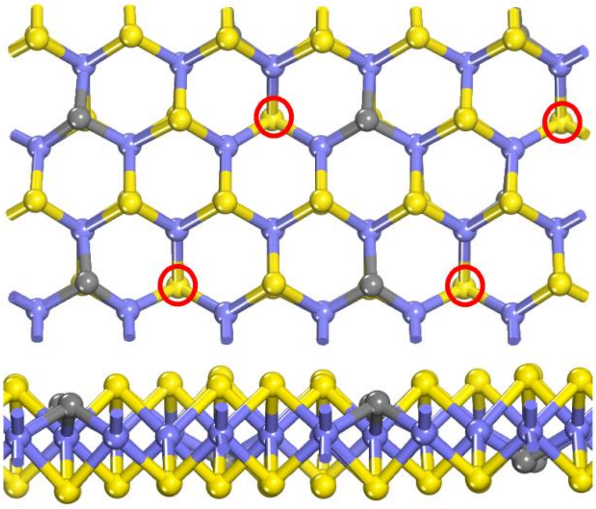

Supplementary Figure 16. Structure analysis of C-MoS_2 with sulfur vacancies. Top-view and side-view structures of C-MoS_2. The red circles denote the vacancy positions.

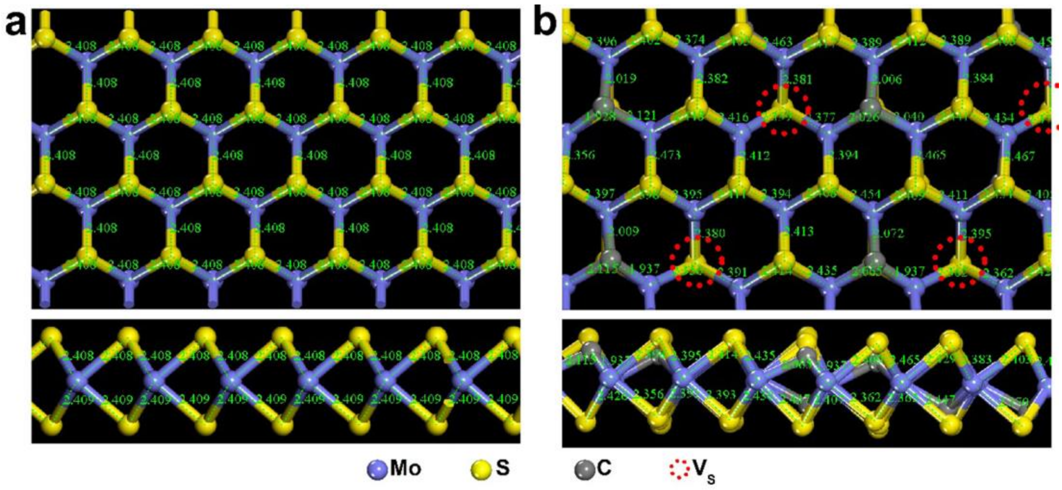

Supplementary Figure 17. Structure and bond length information. The top (upper) and side (lower) views of MoS_2 (a) and C-MoS_2 (b) with detailed Mo-S and Mo-C bond lengths, respectively. The dashed circles denote the vacancy position.

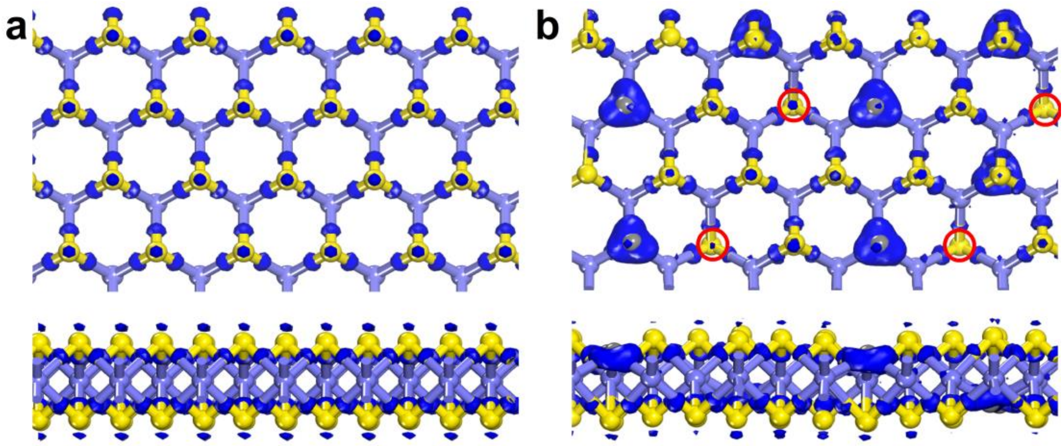

Supplementary Figure 18. Electron density difference analysis of MoS_2 and C-MoS_2. Top-view and side-view electron density differences for (a) MoS_2 and (b) C-MoS_2. The red circles denote the vacancy positions.

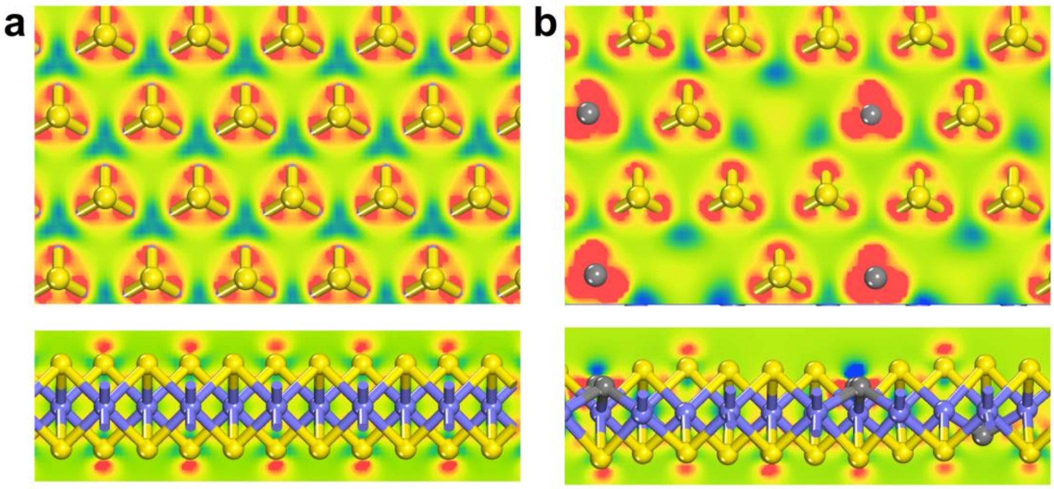

Supplementary Figure 19. Electron density difference analysis of sliced MoS_2 and C-MoS_2. Slices of top-view and side-view electron density differences of (a) MoS_2 and (b) C-MoS_2.

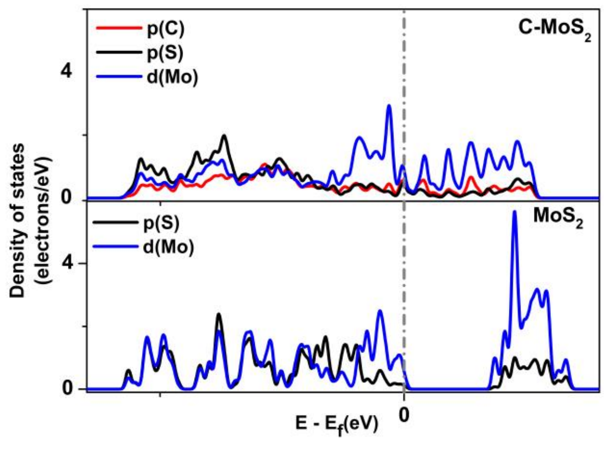

Supplementary Figure 20. Projected DOS (PDOS) analysis. PDOS of valence electrons of the involved elements in MoS_2 and C-MoS_2, respectively.

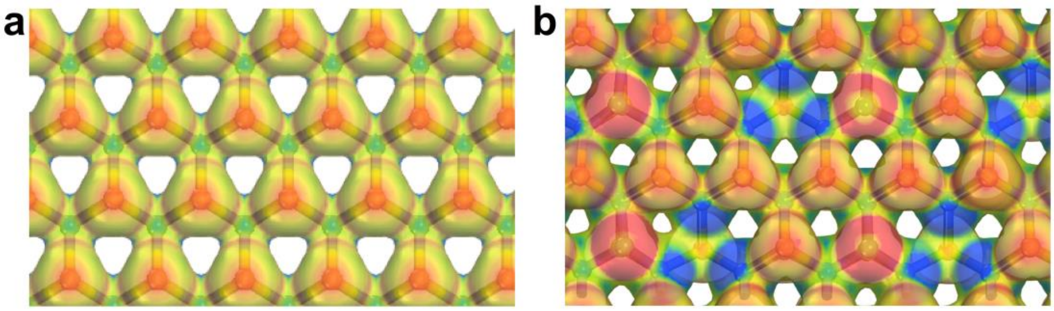

Supplementary Figure 21. Electrostatic potential analysis. Top-view electrostatic potentials of (a) MoS_2 and (b) C-MoS_2.

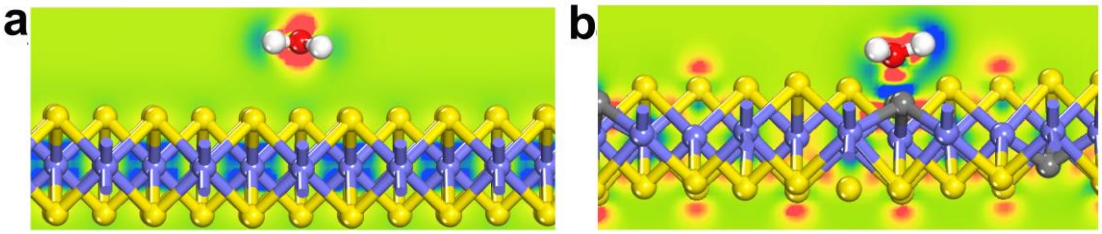

Supplementary Figure 22. Electron density difference analysis of MoS_2 and C-MoS_2 with water adsorption. Slices of side-view electron density difference for water adsorption on the basal plane of (a) MoS_2 and (b) C-MoS_2.

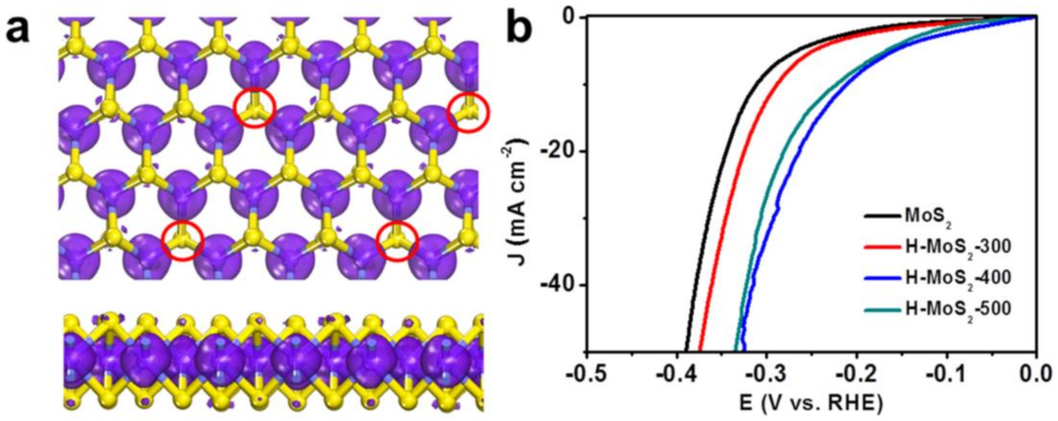

Supplementary Figure 23. Structure analysis and HER properties of MoS_2 with sulfur vacancies. (a) The top and side views of empty orbitals close to the Fermi level of MoS_2 with only vacancies. The red circles denote the vacancy positions. (b) The LSV curves of MoS_2 synthesized on carbon cloth by thermal sulfurization of ammonium molybdate and the H_2 treated counterparts at different temperatures.

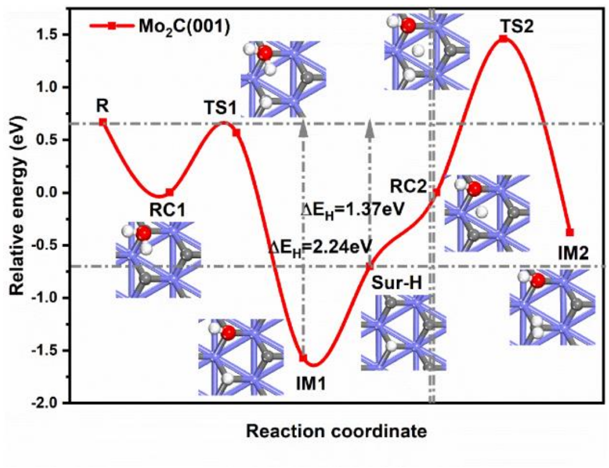

Supplementary Figure 24. The catalytic pathway on the Mo_2C. The relative energy diagram along the reaction coordinate, including the first (left panel) and second (right panel) water dissociation process on the (001) plane of Mo_2C, respectively. R: Reactant, RC: Reactant Complex, TS: Transition State, IM: Intermediate.

Supplementary Table 1. The Mo 3d XPS peak fitting parameters for Mo 3d spectra.

Samples	Binding energy $3d_{5/2}$ ($3d_{3/2}$)	Assignment	FWHM $3d_{5/2}$ ($3d_{3/2}$)
C-MoS_2	229.3 (232.4)	S-Mo-S	1.06 (1.09)
	228.4 (231.5)	S-Mo-C	0.94 (0.96)
MoS_2	229.5 (232.6)	S-Mo-S	1.09 (1.13)
Mo_2C	228.0 (231.1)	C-Mo-C	0.97 (1.02)

Supplementary Table 2. The C1s XPS peak fitting parameters for C 1s spectra.

Samples	Binding energy	Assignment	FWHM
C-MoS_2	283.0	C-Mo	1.66
	284.5	C-C	1.64
	286.0	C-O and C=O	1.77
MoS_2	284.5	C-C	1.68
	285.9	C-O	1.79
Mo_2C	282.9	C-Mo	1.65
	284.5	C-C	1.67
	286.0	C-O and C=O	1.78

Supplementary Table 3. The EXAFS fitting parameters for C-MoS_2 with Mo-C and Mo-O path.

	Path	R (Å)	N	σ^2 ($Å^2$)	ΔE_0 (eV)	R_f (%)
**C-MoS_2**	Mo-C	2.10	1.2	0.0027	2.28	1.1
	Mo-S	2.38	4.9	0.0023		
**C-MoS_2**	Mo-O	2.06	1.0	0.0031	1.13	2.9
	Mo-S	2.39	5.2	0.0029		

Supplementary Table 4. EXAFS fitting parameters for MoS_2 and C-MoS_2.

	Path	R (Å)	N	σ^2 (Å^2)	E_0 (eV)	R_f (%)
**MoS_2**	Mo-S	2.38	6.0	0.0022	2.30	1.3
	Mo-Mo	3.16	6.0	0.0036		
**C-MoS_2**	Mo-C	2.10	1.1	0.0025	2.28	1.9
	Mo-S	2.40	4.8	0.0030		
	Mo-Mo	3.17	6.0	0.0060		

Supplementary Table 5. HER performance of the ever-reported MoS_2-based catalysts in alkaline condition.

Catalysts	Overpotentials at 10 mA cm^{-2} (mV)	Tafel Slopes （mV dec^{-1}）	Electrolyte	Ref.
C-MoS_2	45	46	1.0 M KOH	This work
CoMoS_x	＞ 220	-	PH=13	1
MoS_2/NiCo-LDH on CFP	78	76.6	1.0 M KOH	2
3D macroporous MoS_2 film/Mo foil	184	87	1.0 M KOH	3
$Ni(OH)_2/MoS_2$	80	60	1.0 M KOH	4
MoS_2/Ni_3S_2	110	83.1	1.0 M KOH	5
Co-MoS_2/BCCF-21	48	85	1.0 M KOH	6
N, Mn-MoS_2	66	50	1.0 M KOH	7
Ni doped MoS_2 nanosheets on CC	98	60	1.0 M KOH	8
MoS_2@Ni/CC	91	89	1.0 M KOH	9
Mo–N/C@MoS_2	117	64.3	1.0 M KOH	10

Supplementary Table 6. HER performance of the ever-reported non-precious and precious metal-based catalysts in alkaline condition.

Catalysts	Overpotentials at 10 mA cm^{-2} (mV)	Tafel Slopes mV dec^{-1})	Electrolyte	Ref.
C-MoS_2	45	46	1.0 M KOH	This work
Mo_2C/NCF	100	65	1.0 M KOH	11
Mo_2C−GNR	121	54	0.1 M KOH	12
MoO_{3-x} nanosheets on CC	140	56	0.1 M KOH	13
Mo_2C/CC	140	124	1.0 M KOH	14
MoxC	82	-	0.1 M KOH	15
N,P-doped Mo_2C@C Nanospheres	47	71	1.0 M KOH	16
Mo_2C@NC	60	-	1.0 M KOH	17
$MoNi_4$	15	30	1.0 M KOH	18
Mo_2C@2D-NPC	45	46	1.0 M KOH	19
Mo_2C@NPC	72	52	1.0 M KOH	19
MoP_2NS/CC	67	70	1.0 M KOH	20
Pt_3Ni_3 NWs/C	40	-	1.0 M KOH	21
Ru–MoO_2	29	31	1.0 M KOH	22
$Pt_{13}Cu_{73}Ni_{14}$	148 (η5)	54	1.0 M KOH	23
MoOx/Ni_3S_2/NF	106	90	1.0 M KOH	24
NF–Ni_3Se_2/Ni	203	79	1.0 M KOH	25

Supplementary References

1. Staszak-Jirkovsky, J. et al. Design of active and stable Co-Mo-S_x chalcogels as pH-universal catalysts for the hydrogen evolution reaction. *Nat. Mater.* **15**, 197-203 (2016).

2. Hu, J. et al. Nanohybridization of MoS_2 with layered double hydroxides efficiently synergizes the hydrogen evolution in alkaline media. *Joule* **1**, 383-393 (2017).

3. Pu, Z. H. et al. 3D macroporous MoS_2 thin film: in situ hydrothermal preparation and application as a highly active hydrogen evolution electrocatalyst at all pH values. *Electrochim. Acta* **168**, 133-138 (2015).

4. Zhang, B. et al. Interface engineering: The $Ni(OH)_2/MoS_2$ heterostructure for highly efficient alkaline hydrogen evolution. *Nano Energy* **37**, 74-80 (2017).

5. Zhang, J. et al. Interface Engineering of MoS_2/Ni_3S_2 heterostructures for highly enhanced electrochemical overall-water-splitting activity. *Angew. Chem. Int. Ed.* **55**, 6702-6707 (2016).

6. Xiong, Q. Z. et al. Cobalt covalent doping in MoS_2 to induce bifunctionality of overall water splitting. *Adv. Mater.* **30**, 1801450 (2018).

7. Sun, T. et al. Engineering the electronic Structure of MoS_2 nanorods by N and Mn dopants for ultra-efficient hydrogen production. *ACS Catal.* **8**, 7585-7592 (2018).

8. Zhang, J. et al. Engineering water dissociation sites in MoS_2 nanosheets for accelerated electrocatalytic hydrogen production. *Energy Environ. Sci.* **9**, 2789-2793 (2016).

9. Xing, Z. C., Yang, X. R., Asiri, A. M. & Sun, X. P. Three-dimensional structures of MoS_2@Ni core/shell nanosheets array toward synergetic electrocatalytic water splitting. *ACS Appl. Mater. Interfaces* **8**, 14521-14526 (2016).

10. Amiinu, I. S. et al. Multifunctional Mo-N/C@MoS_2 electrocatalysts for HER, OER, ORR, and Zn-Air batteries. *Adv. Funct. Mater.* **27**, 1702300 (2017).

11. Huang, Y. et al. Mo_2C Nanoparticles dispersed on hierarchical carbon microflowers for efficient electrocatalytic hydrogen evolution. *ACS Nano* **10**, 11337-11343 (2016).

12. Fan, X. J. et al. Atomic H-induce Mo_2C hybrid as an active and stable bifunctional electrocatalyst. *ACS Nano* **11**, 384−394 (2017).

13. Luo, Z. et al. Mesoporous MoO_{3-x} material as an efficient electrocatalyst for hydrogen evolution reactions. *Adv. Energy Mater.* **6**, 1600528 (2016).

14. Fan, M. H. et al. Growth of molybdenum carbide micro-islands on carbon cloth toward binder-free cathodes for efficient hydrogen evolution reaction. *J. Mater. Chem. A* **3**, 16320-16326 (2015).

15. Li, F. et al. Macroporous inverse opal-like Mo_xC with incorporated Mo vacancies for significantly enhanced hydrogen Evolution. *ACS Nano* **25**, 7527-7533 (2017).

16. Chen, Y.-Y. et al. Pomegranate-like N, P-doped Mo_2C@C nanospheres as highly active electrocatalysts for alkaline hydrogen evolution. *ACS Nano* **10**, 8851−8860 (2016).

17. Liu, Y. et al. Coupling Mo_2C with nitrogen-rich nanocarbon leads to efficient hydrogen-evolution electrocatalytic sites. *Angew. Chem. Int. Ed.* **54**, 10752–10757 (2015).

18. Zhang, J. et al. Efficient hydrogen production on $MoNi_4$ electrocatalysts with fast water dissociation kinetics. *Nat. Commun.* **8**, 15437 (2017).

19. Lu, C. B. et al. Molybdenum carbide-embedded nitrogen-doped porous carbon nanosheets as electrocatalysts for water splitting in alkaline media. *ACS Nano* **11**, 3933−3942 (2017).

20. Zhu, W. X. et al. A self-standing nanoporous MoP_2 nanosheet array: an advanced pH-universal catalytic electrode for the hydrogen evolution reaction. *J. Mater. Chem. A* **4**, 7169–7173 (2016).

21. Wang, P., Jiang, K., Wang, K., Yao, J. & Huang, X. Phase and interface engineering of platinum-nickel nanowires for efficient electrochemical hydrogen evolution. *Angew. Chem. Int. Ed.* **55**, 12859 –12863 (2016).

22. Jiang, P. et al. Pt-like electrocatalytic behavior of Ru–MoO_2 nanocomposites for the hydrogen evolution reaction. *J. Mater. Chem. A* **5**, 5475–5485 (2017).

23. Shen, Y., Lua, A. C., Xi, J. Y. & Qiu, X. P. Ternary platinum-copper-nickel nanoparticles anchored to hierarchical carbon supports as free-standing hydrogen evolution electrodes. *ACS Appl. Mater. Interfaces* **8**, 3464–3472 (2016).

24. Wu, Y. Y. et al. Overall water splitting catalyzed efficiently by an ultrathin nanosheet-built hollow Ni_3S_2 based electrocatalyst. *Adv. Funct. Mater.* **26**, 4839–4847 (2016).

25. Cao, J. M., Zhou, J., Zhang, Y. F. & Liu, X. W. Ni_3Se_2 nanoforest/Ni foam as a hydrophilic, metallic, and self-supported bifunctional electrocatalyst for both H_2 and O_2 generations. *Nano Energy* **24**, 103–110 (2016).
